# Notes and News

**Published:** 1892-07-16

**Authors:** 


### Notes and News.

COLLECTED AND COMMUNICATED.

Sir Andrew Clark, Dr. Billings, of Washington, Jonathan Hutchinson, Dr. Gusseron, of Berlin, and Thomas Bryant, received the honorary degree of Doctor of Medicine at the Tercentenary Celebration of the Dublin University on the 6th inst.

The General Election has been made use of by the Society for the Abolition of Compulsory Vaccination as a means to bring their views strongly forward. Eleven resolutions were brought forward at their last meeting, the final one of which ran, "That no candidate (Parliamentary) who declines to pledge himself to the abolition of the compulsory clauses of the Vaccination Acts can be deemed satisfactory." Electors are advised to write to present Members, who have failed to vote against compulsory vaccination, informing them that they will never have their votes again, unless their position as to this question is changed.

Mr. Frederic Walter Beck writes to report that a successful Hospital Saturday collection has been made in connection with the Luton Bute Hospital. This collection has only been started for three years, and has steadily grown in success. This year, for the first time, a committee of ladies assisted in the collection, and so raised £60, so that the amount realised was £197 12s., a large advance on other years. The hospital is now under the charge of a highly skilled Matron in Miss Babcock, formerly of St. Thomas's Hospital, and while it is not yet sufficiently extensive or wealthy to maintain a house surgeon, the principal medical men of the town are most devoted in their services. The result of good management and attention has been to make the hospital so popular that, in consequence of the increasing numbers seeking admission, the Committee, though sadly in need of funds, have been obliged to arrange for an extension at a cost of over £600. This work is now being carried out, and a special appeal is being made for funds to meet the outlay.

The North-West London Hospital which has this year been so fortunate in finding good friends, has in connection with it a special source of income, to which we desire to draw our readers' attention, as we cannot help thinking the scheme has great possibilities of development and general application. This scheme is a collection of annual subscribers of half-a-crown, by a gentleman interested in the institution. The collection bears the title of "The Half-Crown Fund." In the case in question the fund is proving a great success, and one energetic friend at each hospital undertaking such a collection would not only benefit the institution financially, but would also multiply the interest in it to an extent at first not to be realised. We will assume that each householder sets aside a certain definite sum towards works of charity, but this is probably not the case with the other members of the household, many of whom would shrink from contributing so small a sum in any other manner. The collector need not shrink from pleading for so small a sum, and if the object of the collection is one of the metropolitan hospitals, he may extend his labours to country friends, considering how large a number of patients from the country yearly enter the London hospitals.

Monsieur Charcot, in an article in the *Progres Medical*, gives a most interesting account of the phenomenal calculator, Jacques Inaudi. Inaudi's first occupation was tending sheep at his home in Piedmont. He gave signs of his extraordinary capacity when, at six years old, he commenced arithmetic with his brother, learning orally, being neither able to read nor write. In 1880, when twelve years old, and still quite untutored except in the matter of arithmetic, he was brought before the Anthropological Society of Paris. He is now twenty-four years of age, fairly educated, small of stature, robust, and quite normal in appearance. His extraordinary faculties have been lately the subject of special scientific ininquiry. It is granted that memory undoubtedly plays the most important part in effecting the wonderful results of his calculations. The world has seen many unusually rapid calculators. Inaudi has distanced them all. Mondeux, his predecessor, succeeded in doing a difficult calculation in five minutes; Inaudi accomplished the same elaborated in fifty-nine seconds. It would appear that his *modus operandi* is also unique. Calculators who have gone before him have admittedly relied upon a mental vision of the figures. Bidder, in his memoirs, stated that he could not conceive it possible to accomplish any process without mental photography. Inaudi, on the contrary, declares himself hampered when confronted with written figures. He must in all cases articulate them, as he depends on oral vibrations for his impressions. He declares that the sound of each figure is reproduced, and that the impression is so strong that the sounds continue to repeat themselves mentally to him for hours after they have been heard. His memory is unusually retentive of figures alone. Mons. Charcot holds that especial activity of the memory in one direction causes isolation and a possibility of independent development in one direction. The inquiry has not resulted in any hereditary tendency being discovered for Inaudi's unusual faculty.

